# The Complete Genome Phylogeny of Geographically Distinct Dengue Virus Serotype 2 Isolates (1944-2013) Supports Further Groupings within the Cosmopolitan Genotype

**DOI:** 10.1371/journal.pone.0138900

**Published:** 2015-09-28

**Authors:** Akhtar Ali, Ijaz Ali

**Affiliations:** Department of Biological Science, The University of Tulsa, Tulsa Oklahoma, 74104, United States of America; University of Padua, ITALY

## Abstract

Dengue virus serotype 2 (DENV-2) isolates have been implicated in deadly outbreaks of dengue fever (DF) and dengue hemorrhagic fever (DHF) in several regions of the world. Phylogenetic analysis of DENV-2 isolates collected from particular countries has been performed using partial or individual genes but only a few studies have examined complete whole-genome sequences collected worldwide. Herein, 50 complete genome sequences of DENV-2 isolates, reported over the past 70 years from 19 different countries, were downloaded from GenBank. Phylogenetic analysis was conducted and evolutionary distances of the 50 DENV-2 isolates were determined using maximum likelihood (ML) trees or Bayesian phylogenetic analysis created from complete genome nucleotide (nt) and amino acid (aa) sequences or individual gene sequences. The results showed that all DENV-2 isolates fell into seven main groups containing five previously defined genotypes. A Cosmopolitan genotype showed further division into three groups (C-I, C-II, and C-III) with the C-I group containing two subgroups (C-IA and C-IB). Comparison of the aa sequences showed specific mutations among the various groups of DENV-2 isolates. A maximum number of aa mutations was observed in the NS5 gene, followed by the NS2A, NS3 and NS1 genes, while the smallest number of aa substitutions was recorded in the capsid gene, followed by the PrM/M, NS4A, and NS4B genes. Maximum evolutionary distances were found in the NS2A gene, followed by the NS4A and NS4B genes. Based on these results, we propose that genotyping of DENV-2 isolates in future studies should be performed on entire genome sequences in order to gain a complete understanding of the evolution of various isolates reported from different geographical locations around the world.

## Introduction

Dengue is an emerging and re-emerging infectious disease caused by a mosquito-borne, single-stranded, positive-sense RNA virus named the dengue virus (DENV) (genus *Flavivirus*, family *Flaviviridae*). DENV has four antigenically related but genetically different serotypes: DENV-1, DENV-2, DENV-3 and DENV-4. The genome of DENV is approximately 11 kb, containing a single open reading frame (ORF) flanked by 5´ and 3´ UTRs. Translation of the ORF produces a large polyprotein that is cleaved into 10 mature proteins. The N-terminal of the polyprotein encodes three structural proteins: capsid (C), premembrane/membrane (PrM/M), and envelope (E), as well as seven non-structural (NS) proteins: NS1, NS2A, NS2B, NS3, NS4A, NS4B, and NS5, which are flanked by 5´ and 3´-non-translated regions (5´ NTR/3´NTR) [1, 2].

The first reported epidemics of dengue fever occurred from 1779–1780 in Asia, Africa, and North America; however, the first global pandemic began after World War II [3]. Over the next 60 years, the geographic distribution of dengue expanded considerably, and now all four serotypes of the virus are circulating in Asia, Africa, and the Americas [4].

Different DENV serotypes (DENV-1, DENV-2, DENV-3 and DENV-4) are important with respect to their association with sylvatic cycles, DF outbreaks, and low to high transmission to humans, as well as DHF or dengue shock syndrome (DSS) [1]. Our study focuses solely on the DENV-2 isolates as this serotype is more prevalent worldwide and has been associated with a number of epidemics. In addition, large numbers of complete genome sequences of DENV-2 from diverse geographical locations are available in GenBank, as compared to serotypes 1, 3, and 4. DENV-2 is also the most frequently circulating serotype in Pakistan, as reported in several outbreaks from 2005–2013 in Pakistan, and 10 complete genome sequences of Pakistan DENV-2 isolates are available in the GenBank database. Therefore, we restricted this study strictly to DENV-2 isolates, and future studies will be focused on the remaining serotypes, depending on the availability of complete genome sequences from different countries. The evolutionary history of dengue viruses is recent, but DENV-2 is believed to have emerged 120 to 215 years ago [5, 6, 7, 8]. DENV-2 has been linked to severe epidemics of DHF in various geographical regions of the world [9, 10, 11, 12, 13, 14, 15, 16, 17, 18, 19, 20, 21]. Recently, severe epidemics of DENV-2 caused high morbidity and mortality in South Asia [11, 12, 22]. Being a *Flavivirus*, DENV-2 is prone to rapid mutation as it replicates in known hosts, such as human beings and mosquitoes of the *Aedes* genus. It has also been recently detected in bats [23].

Reverse transcription polymerase chain reaction (RT-PCR) and real-time PCR have been used for many years for the identification of DENV serotypes [24]. However, increasing viral intra-genetic diversity requires a more effective method for genotype identification [1]. Phylogenetic analysis based on individual gene sequences has, therefore, recently proved useful for the genotyping of DENV-2 [1, 7, 25]. Based on envelope gene sequences, DENV-2 has been divided into five distinct genotypes: American, Asian-American, Asian-I, Asian-II, and Cosmopolitan [7]. Various genotypes of DENV-2 have been the causative agents of the worst epidemics, resulting in high morbidity and mortality in a number of different countries [9, 10, 11, 12, 13, 14, 16, 17, 18, 19, 20, 21, 26].

A plethora of phylogenetic studies have previously used partial DENV-2 genomic sequences of the capsid, PrM, envelope, or other genes for finding viral factors responsible for pathogenicity and evolutionary and transmission trends [8, 9, 11, 12, 13, 22, 27, 28, 29, 30, 31, 32, 33]. Several studies have used a partial or truncated 5´ or 3´ C-PrM region for conducting phylogenetic analysis [11, 29, 33]. A few studies have recently used ORF or complete genome sequences for finding the phylogenetic relationship of DENV-2 isolates restricted to a particular region or country [10, 13, 22, 25, 34]. Partial genomic sequences are frequently being used for evolutionary analysis of DENV-2. Thus, there is a need to determine whether focusing on a particular gene or utilizing entire genome sequences is best suited for the genotyping of DENV-2 isolates worldwide.

In this study, we compared the complete genome sequences of 50 DENV-2 isolates (isolated from 1944 to 2013), including 10 Pakistan isolates, in order to determine genetic diversity, selection pressure on particular genes, and evolutionary distances over time.

## Material and Methods

### Source of sequences and phylogenetic tree

Entire genome sequences of 50 DENV-2 isolates (Table 1) were selected and retrieved from the GenBank NCBI database, as they were representative of diverse geographical locations in 19 different countries spanning South Asia, Southeast Asia, the Far-East, Africa, Australia, North America, and South America. Minor and major dengue outbreaks had previously been recorded in these regions and whole-genome sequences of DENV-2 isolates had been characterized. In addition, these 50 DENV-2 isolates were divided into seven temporal classes, which included one isolate each from 1944 and 1964, five isolates from 1970–1980, six isolates from 1981–1990, five isolates from 1991–2000, 24 isolates from 2001–2010, and eight isolates from 2011–2013. (The number of DENV-2 isolates selected fluctuates decade by decade according to the availability of complete genome sequences in the GenBank database). We randomly selected representative isolates of DENV-2 from South and North America that included isolates from Columbia, Peru and the USA. Therefore, we did not include isolates from Brazil and Venezuela. Previous studies have used a total of either 22 [1] or nine [25] DENV-2 isolates for classification and evolutionary analysis, including the New Guinea C isolate (NGC), which is generally used as the standard for temporal analysis targeting evolutionary divergence. We included representative sequences from a number of geographical regions in order to have a broader picture and clearer understanding of the spatio-temporal evolution and classification of DENV-2. Although there are temporal standards for analysis, there is no consensus on criteria for selecting spatial representative sequences.

**Table 1:** Locations and names of DENV-2 isolates collected worldwide during 1944–2013.

Country	Name of isolate	Genome (bp)	Year	Nucleotide Accession	Protein Accession	Reference
Australia	TSV01	10723	1993	AY037116	AAK67712	[46]
Brunei	DS09-280106	10709	2006	EU179859	ABW06614	[47]
	DS31-291005	10709	2005	EU179857	ABW06612	[47]
Burkina Faso	1349	10723	1983	EU056810	ABW74619	[48]
China	GD01/03	10723	2003	FJ196853	ACN54392	[49]
	44	10723	1989	AF204177	AAF18446	Direct submission
	43	10723	1987	AF204178	AAF18447	Direct submission
	China 04	10723	1985	AF119661	AAD18036	Direct submission
	FJ11/99	10723	1999	AF359579	AAK49562	Direct submission
	FJ-10	10723	2000	AF276619	AAF86463	Direct submission
China/India	QHD13CAIQ	10723	2013	KF479233	AHA42535	[32]
Colombia	CO/BID-V3358	10667	1986	GQ868592	ACW82882	Direct submission
Fiji	FJ/UH21/1971	10714	1971	HM582099	ADM26218	[50]
Guam	GU/BID-V2950	10967	2001	HM488257	ADK26435	Direct submission
Guatemala	American Asian	10725	2009	HQ999999	AER45462	[34]
India	GWL18	10670	2001	DQ448231	ABE02262	Direct submission
	IN/BID-V2961	10669	2006	FJ898454	ACQ44493	Direct submission
	Od2112	10670	2011	JQ955624	AFZ40226	[22]
	RR44	10670	2009	JQ955623	AFZ40225	[22]
	1392	10670	2009	JX475906	AFZ40227	[22]
Indonesia	1016DN	10723	1975	GQ398258	ADK37474	[51]
	1017DN	10723	1976	GQ398259	ADK37475	[51]
	1070DN	10723	1976	GQ398260	ADK37476	[51]
	98900663DHF	10723	1998	AB189122	BAD42415	Direct submission
	BA05i	10723	2004	AY858035	AAW51406	[52]
	1022DN	10724	1975	GQ398268	ADK37484	[51]
New Guinea	NGC	10724	1944	AF038403	AAC59275	[53]
Pakistan	Pak-L-2011	10723	2011	KF041234	AHC72406	[33]
	Pak-L-2011	10723	2011	KF041232	AHC72404	[33]
	Pak-K-2009	10723	2009	KF041237	AHC72409	[33]
	Pak-K-2009	10723	2009	KF041235	AHC72407	[33]
	Pak-M-2011	10723	2011	KF041233	AHC72405	[33]
	PakL-2013	10629	2013	KJ010186	AHM25910	Direct submission
	Pak—L-2011	10629	2011	KJ010185	AHM25909	Direct submission
	Pak-L-2010	10629	2010	KF360005	AHA80987	Direct submission
	Pak-L-2008	10723	2008	KF041236	AHC72408	[33]
Peru	PE/NFI1159	10723	2010	KC294223	AGX15388	[54]
	PE/IQA 2080	10723	2010	KC294221	AGX15386	[54]
Singapore	SG/D2Y98P-PP1	10723	2009	JF327392	AEI29060	[56]
	SG/05K3295DK1	10723	2005	EU081177	ABW82013	[57]
Sri Lanka	LK/BID-V2421	10629	2003	GQ252676	ACS32038	Direct submission
	LK/BID-V2422	10628	2004	GQ252677	ACS32039	Direct submission
	LK/BID-V2416	10628	1996	FJ882602	ACQ44409	Direct submission
Taiwan	1222-DF-06	10671	2002	DQ645546	ABG29081	Direct submission
	TW/BID-V5056	10615	2008	HQ891024	AEH59344	Direct submission
Thailand	TH/BID-V3357	10678	1964	GQ868591	ACW82881	Direct submission
USA	US/BID-V5412	10484	2007	JF730050	AEH59345	Direct submission
	US/BID-V5055	10488	2008	JN796245	AET72454	Direct submission
	IQT1797	10674	1998	AF100467	AAD32962	[55]
Vietnam	VN/BID-V735	10678	2006	EU482672	ACA48939	Direct submission
USA-DENV1	US/Hawaii/1944	10734	1944	EU848545	ACF49259	Direct submission

Complete sequences of each isolate were manually fragmented into 13 segments that included 5´UTR, C, PrM/M, E, NS1, NS2A, NS2B, NS3, NS4A, NS4B, NS5, 3´UTR and the complete ORF. Nucleotide sequences of all individual genes, 5´ and 3´ UTRs, ORFs, and whole-genome nt or aa sequences were aligned using the Clustal X program [35]. Phylogenetic analysis was performed with the MEGA5 program [36] using the maximum-likelihood (ML) method, based on the general time reversible (GTR) or GTR+I+G nucleotide substitution models. The robustness of all ML trees was tested with 1000 bootstrap replications.

### Bayesian MCMV evolutionary analysis

**Fig 1:**
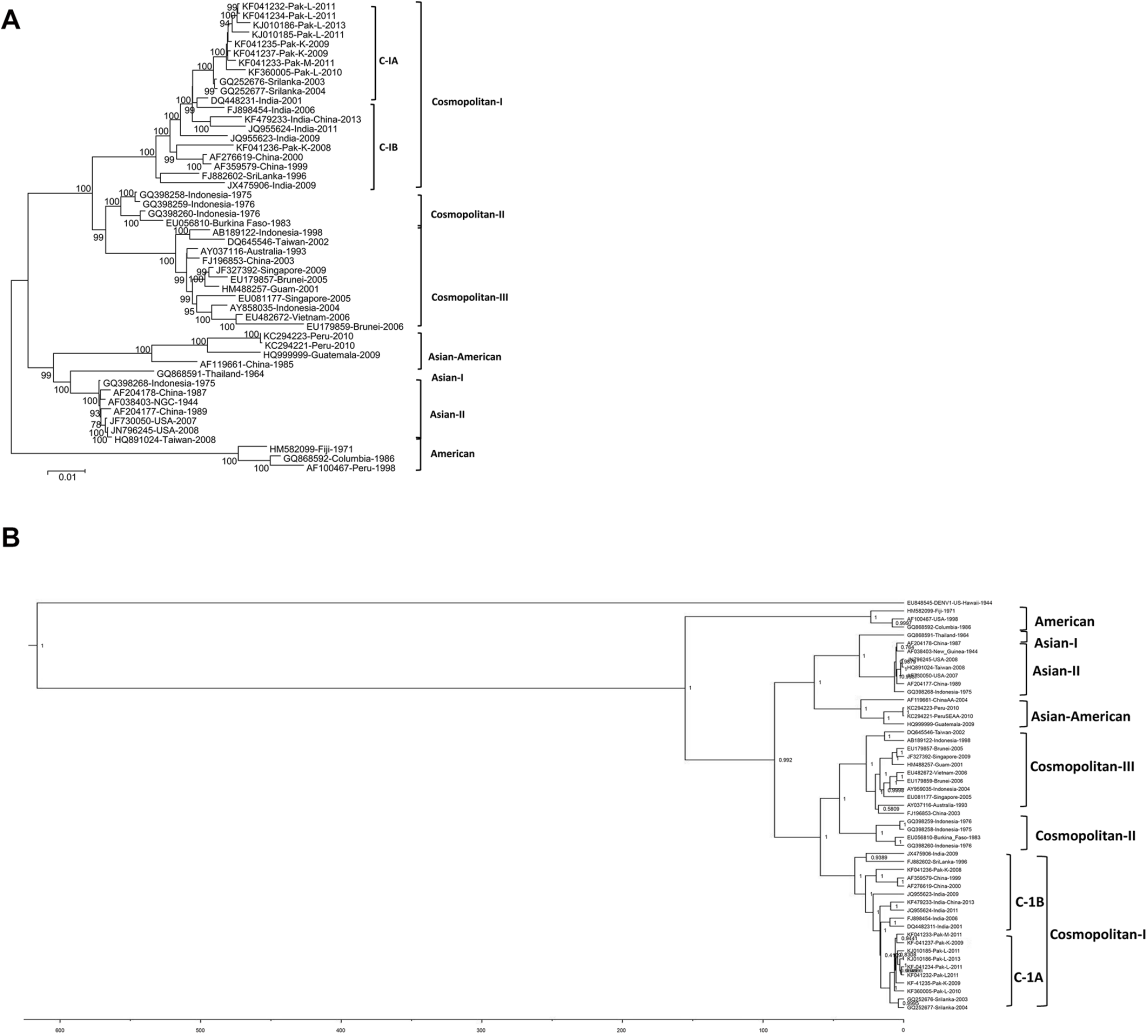
Phylogenetic analysis of DENV-2 complete genome nucleotide sequences. (A) Maximum-likelihood trees were constructed using MEGA V5.05 software with bootstrap support of 1000 replicates. All nucleotide sequences were downloaded from the GenBank database for analysis (Table 1). The phylogenetic tree was constructed using the General Time Reversible (GTR) model. (B) Bayesian Maximum Clade Credibility tree of the 50 DENV-2 isolates. Seven groups including five major genotypes were identified. In the Cosmopolitan genotype, there were three groups (C-I, C-II and C-III) while C-I was sub-grouped into C-IA and C-IB. The DENV-1 US/Hawaii isolate was used as an out-group.

After phylogenetic analysis using ML trees, the Bayesian Markov chain Monte Carlo (MCMC) approach, as implemented in the BEAST package v1.8.2 (Available online at http://tree.bio.ed.ac.uk/software/) was used to analyze the complete genome sequences of the 50 DENV-2 isolates. The data were analyzed using the Bayesian Skyline speciation model and the GTR+G model of evolution with empirical base frequencies and lognormal relaxed clock with 20 million generations. We set a burn-in of 20% for posterior probabilities and then examined the results using Tree Annotator followed by TRACER v1.6 programs from the BEAST package. The tree was visualized in Fig tree v1.4.2. The complete genome of the DENV-1 US/Hawaii isolate (Table 1) was also downloaded from GenBank and was used as an out-group in the BEAST analysis (Fig 1B).

### Evolutionary distances among DENV-2 isolates

Once the sequences were aligned for either the whole-genome or individual genes, those files were used in the MEGA5 program to determine first the best model and then the overall evolutionary distances among the DENV-2 isolates (Table 2). Bootstrap resampling analyses were performed using 1000 replicates.

**Table 2:** Evolutionary distances among the complete genome or individual genes of 50 DENV-2 isolates collected from various countries during 1944 to 2013.

Genome/gene	Size (Nucleotides)	Size (Amino Acids)	Mean Distance
Complete genome	10723	3391	0.06036 ± 0.00124
ORF	10272		0.06103 ± 0.00136
5´-UTR	Minimum (64)		
	Maximum (96)		
Capsid	342	114	0.05316 ± 0.00728
PrM/M	498	166	0.05855 ± 0.00689
Envelope	1485	495	0.05316 ± 0.00740
NS1	1056	352	0.06114 ± 0.00418
NS2A	654	218	0.08272 ± 0.00768
NS2B	390	130	0.06500 ± 0.00819
NS3	1854	618	0.06113 ± 0.00320
NS4A	450	150	0.06866 ± 0.00782
NS4B	744	248	0.06511 ± 0.00580
NS5	2703	901	0.05800 ± 0.00275
3´ UTR	Minimum (120)		0.03885 ± 0.0115
	Maximum (643)		

### Determination of group-specific amino acid patterns

Amino acid sequences of the 50 DENV-2 isolates (Table 1) were retrieved from the GenBank NCBI data base and manually fragmented into individual protein sequences. All the respective amino acid sequences were aligned using the Clustal X program, as above. Group-specific amino acid patterns were determined manually from the aligned sequences in the respective genotype groups.

## Results

The size of the genome varied from 10484 to 10724 nucleotides among the 50 DENV-2 isolates with a diverse geographical background encompassing South Asia, East Asia, Africa, Australia, and North and South America. However, the ORF size (10240) was the same among all the DENV-2 isolates (Table 1). The main differences in the sizes of the genomes were due to variations in the length of 5´ or 3´ UTRs rather than in the length of the ORFs.

### Phylogenetic analysis based on complete genome nucleotide sequences

Using the ML method, phylogenetic analysis of the 50 DENV-2 isolates showed seven main groups containing five previously defined genotypes (Cosmopolitan, American, Asian-American, Asian-I, and Asian-II). Out of these 50, 35 DENV-2 isolates were clustered in the Cosmopolitan group, three in the American, four in the Asian-American, seven in the Asian-II, and one isolate in the Asian-I group (Fig 1A). Phylogenetic analysis showed three main groups within the Cosmopolitan genotype, which were designated as C-I, C-II, and C-III, with a bootstrap support of 100 (Fig 1A). The C-I group was further divided into two subgroups, which were named Cosmopolitan IA (C-IA) and Cosmopolitan IB (C-IB).

The two Sri Lankan and all the Pakistani isolates except one (Accession # KF041236) clustered in C-IA, which is a distinct subgroup but closely related to C-IB, that contains 10 DENV-2 isolates from China, India and Sri Lanka. The only other Pakistani DENV-2 isolate, reported in 2008 from Karachi (Accession # KF041236), clustered with isolates in the C-IB subgroup and was different from the rest of the Pakistani isolates, which clustered in C-1A. This same DENV-2 isolate from Karachi, Pakistan, was more closely related to the Chinese isolates (Fig 1A) than to the other Pakistani isolates.

The second main group of the Cosmopolitan genotype (C-II) contained four isolates, three Indonesian DENV-2 isolates from 1975/76 and one being from Burkina Faso originally identified in 1943. Cosmopolitan III (C-III) also made up a distinct major group containing 11 isolates from East Asia, Southeast Asia, Africa and Australia. Both the C-II and C-III groups were supported by a bootstrap value of 100 (Fig 1A).

The phylogeny based on the entire genome sequences revealed that the 8 Asian isolates clustered into two distinct groups (Asian-I and Asian-II), and were genetically closer to the Asian-American DENV-2 genotypes (Fig 1A).

### BEAST analysis

The phylogenetic tree reconstructed by Bayesian analysis is shown in Fig 1B. The Bayesian tree topology was highly similar to that recovered using ML methods (Fig 1A). The phylogeny also showed that the Cosmopolitan genotype has three main groups: C-I, C-II, and C-III. The C-I group contains subgroups C-IA and C-IB, and the distribution of isolates is exactly the same as obtained from the ML trees (Fig 1A). The posterior probability of each node is denoted by 1 (100%). These results shows that two different methods confirm our conclusions that the Cosmopolitan genotype should be further divided into three groups. The Pakistani DENV-2 isolates are estimated to have emerged in the last 10 years (Fig 1B).

### Phylogenetic analysis based on amino acid sequences

**Fig 2:**
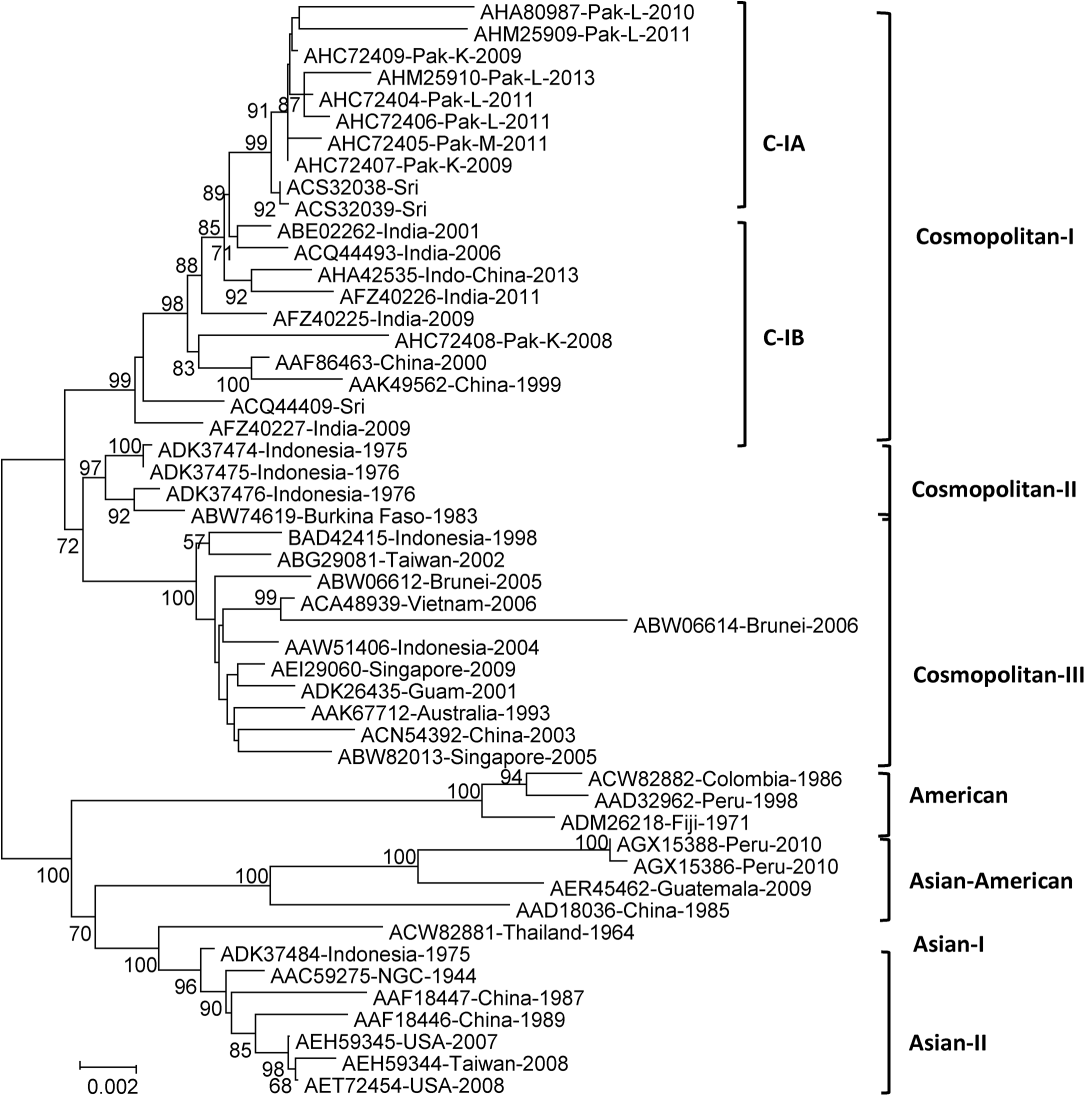
Phylogenetic maximum-likelihood trees of DENV-2 complete genome amino acid sequences. Trees were constructed using MEGA V5.05 software with bootstrap support of 1000 replicates. All amino acid sequences were downloaded from the GenBank database for analysis, and the respective DENV-2 isolates with their accession numbers are listed in Table 1.

Phylogenetic analysis of the 50 DENV-2 isolates based on complete genome amino acid sequences also showed seven distinct main groups (Fig 2). The Cosmopolitan genotype fell into three major groups (C-I, C-II and C-III) with >90 bootstrap support, while C-I was subdivided into C-IA and C-IB. This matches the results of both genotype analyses.

### Phylogenetic analysis based on ORFs

**Fig 3:**
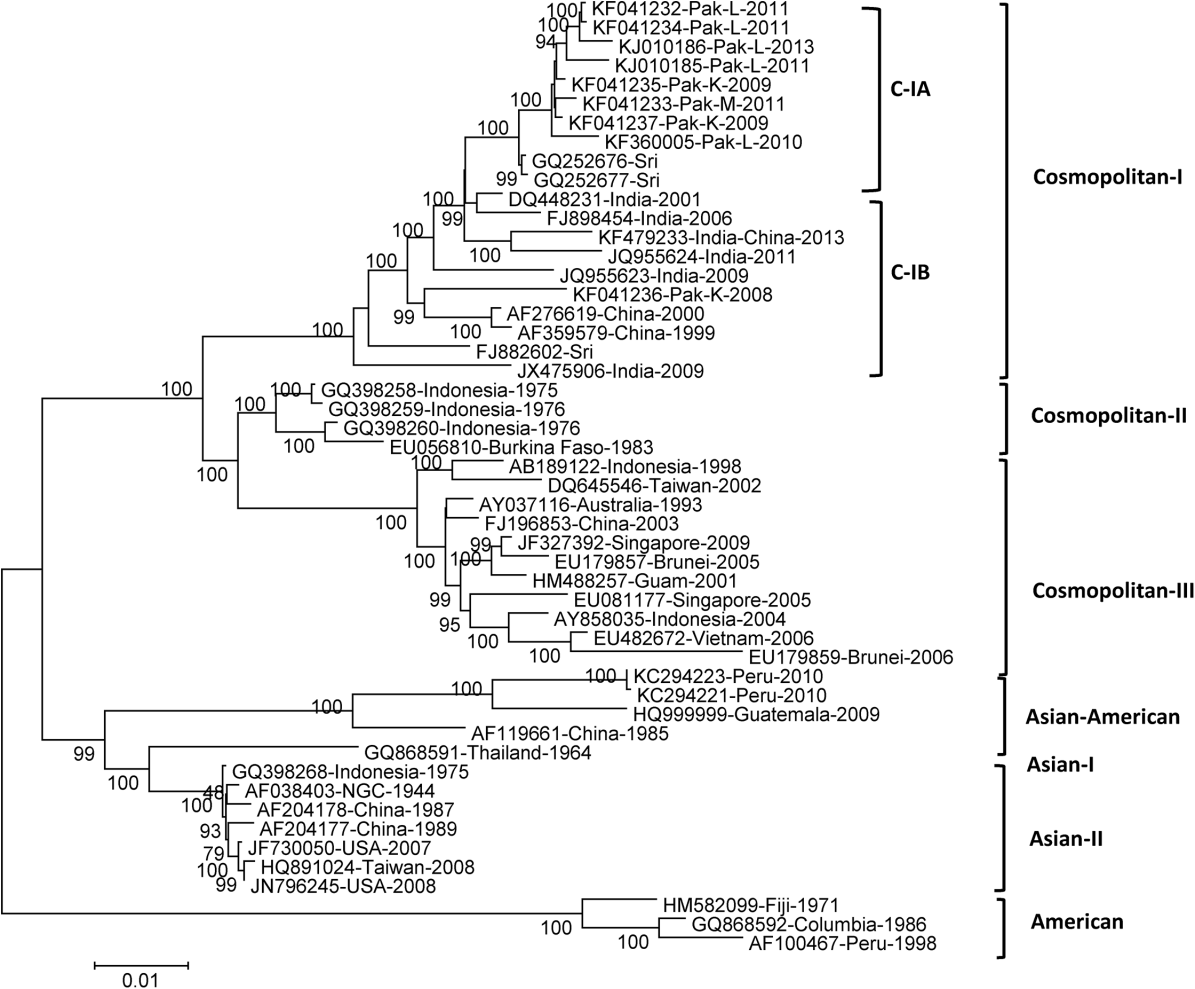
Phylogenetic maximum-likelihood trees of DENV-2 ORF nucleotide sequences. Trees were constructed using MEGAV5.05 software with bootstrap support of 1000 replicates. All sequences of the ORF were manually separated from the whole-genome sequences that were downloaded from the GenBank database for analysis. The phylogenetic tree was constructed using the GTR model.

Phylogenetic analysis of all 50 isolates based on complete ORFs showed almost exactly the same results as obtained on the basis of entire genome nucleotide sequences (Fig 3). The topology of the ORF-based tree was highly similar to the complete genome nt or aa trees and showed the same distribution of DENV-2 types (Fig 3).

### Group-specific patterns of aa mutations among various genotypes

When amino acids sequences were aligned from the 50 DENV-2 isolates, group-specific patterns of aa mutations were observed among the various groups. The nature of specific patterns common to various groups (Table 3) and the number of patterns of mutations found in individual groups (Table 4) revealed that the highest number of specific patterns (n = 24) was present in the American isolates, followed by the Asian-American (n = 10), C-IA (n = 7) and C-III (n = 6). The lowest number of type-specific patterns was observed in the case of the Asian-American isolates. No type-specific aa mutations were observed in C-IB, C-II, or Asian-I, alone; however, C-IB shared 14 group-specific mutations with C-IA.

**Table 3:** Type-specific amino acid mutations among the various groups of DENV-2 isolates.

Cosmopolitan						Asian-II		Asian-I	Asian/American		American	
C-IA		C-IB	C-II	C-III								
AA	Mut	-	-	AA	Mut	AA	Mut		AA	Mut	AA	Mut
130	R→K	**-**	**-**	108	L→M	1266	N→K	**-**	130	R→G	275	V→I
506	T→I	-	-	143	D→N	1301	V→A	**-**	1231	T→A	351	A→D
862	T→S	-	-	892	T→A	1662	R→K	**-**	1236	V→A	361	S→I
1243	I→T	-	-	906	Q→H	1820	N→S	**-**	1444	I→V	670	N→D
1301	V→I	-	-	1047	K→R	2355	L→F	**-**	1504	K→R	848	P→S
2891	T→A	-	-	1298	I→T				1536	K→R	887	K→R
3352	K→R	-	-						2418	V→I	1032	N→S
									2647	V→I	1242	T→S
									2736	R→K	1407	E→D
									3327	I→V	1842	A→T
											1856	K→R
											1941	K→R
											2042	I→T
											2043	K→R
											2444	I→V
											2670	C→V
											2684	K→V
											3044	V→I
											3049	E→A
											3129	T→S
											3138	L→V
											3219	V→T
											3291	R→S
											3310	Q→L
Total	7	0	0		6		5			10		52

**Footnote:** AA: Amino acid, C-IA: Cosmopolitan IA, C-IB: Cosmopolitan IB, C-II: Cosmopolitan II, C-III: Cosmopolitan III, Mut; mutation

**Table 4:** Total number of type-specific amino acid mutations in individual genes among various genotypes of DENV-2 isolates.

	Cosmopolitan				Asian-II	Asian-I	Asian/American	American	Total
	C-I		C-II	C-III					
	C-IA	C-IB							
No. of isolates	10	10	4	11	8	0	4	3	50
Capsid	0	0	0	1	0	0	0	0	1
PrM/M	1	0	0	1	0	0	1	1	4
E	1	0	0	0	0	0	0	3	4
NS1	1	0	0	3	0	0	0	3	7
NS2A	2	0	0	1	2	0	2	1	8
NS2B	0	0	0	0	0	0	1	1	2
NS3	0	0	0	0	2	0	2	3	7
NS4A	0	0	0	0	0	0	0	2	2
NS4B	0	0	0	0	1	0	1	1	3
NS5	2	0	0	0	0	0	3	9	14
**Total**	**7**	**0**	**0**	**6**	**5**	**0**	**10**	**24**	**52**

The subgroup C-IA contained seven distinct patterns of aa mutations, distinguishing it from isolates in C-IB and the rest of the DENV-2 isolates used for comparison. Although subgroup C-II shared some aa substitutions with C-III, the latter had six unique patterns that clearly distinguished the isolates in C-II from those in C-III (Tables 3 & 4). The most frequent pattern found at different positions among the isolates was K→R followed by V→I (Table 3). The fewest number of group-specific patterns of mutations (2 each) was found in the C and NS4A genes, while the highest was found in NS5 (n = 14), followed by NS2A (n = 8), with n = 7 in the case of the NS1 and NS3 genes (Table 3). The NS2A gene was the only one that contained group-specific mutations in a majority of the groups (Table 4), which effectively differentiated the DENV-2 genotypes. The nature of the type-specific mutations observed in the NS2A gene is given in Table 3.

### Evolutionary distances across the entire genome, ORFs or individual genes of DENV-2

Evolutionary distances across the amino acids sequences of the entire genome, the ORFs, the individual genes and the UTRs were determined. Minimum distances were noted in the 5´ UTR (0.02355 ± 0.05397) and 3´ UTR (0.03885 ± 0.0115), while maximum distances were found in the NS2A gene (0.08272 ± 0.00768), followed by the NS4A (0.06866 ± 0.00782) and NS4B genes (0.06511 ± 0.00580). Evolutionary distances observed in NS5 were less than all other non-structural genes and were most similar to those observed in structural genes (Table 2).

### Phylogenetic analysis based on individual genes

**Fig 4:**
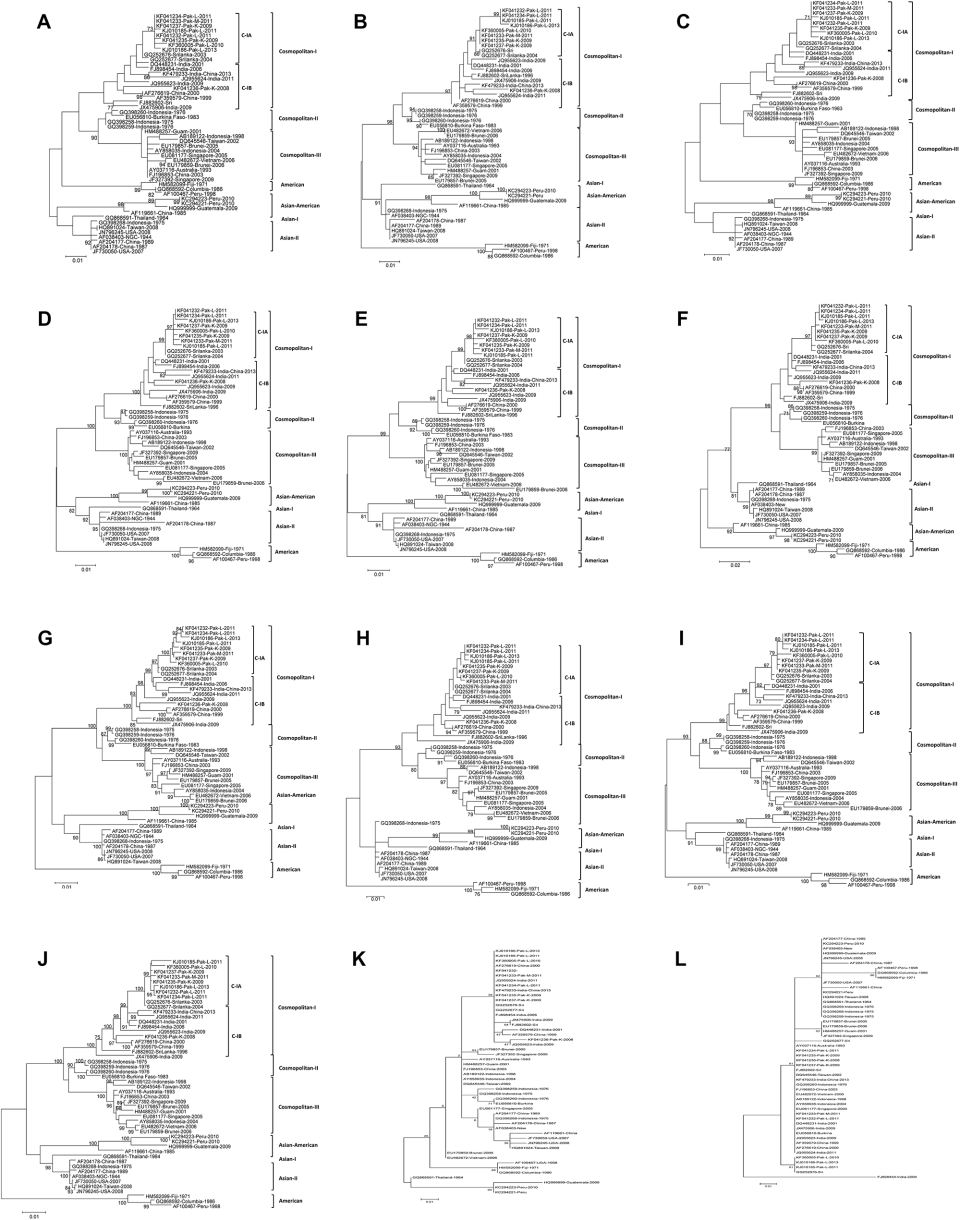
Phylogenetic maximum-likelihood trees of DENV-2 individual gene nucleotide sequences. All sequences of the individual genes were manually separated from the complete genome sequences that were downloaded from the GenBank database for analysis. The phylogenetic trees were constructed using the GTR model. (A) C gene; (B) PrM/M gene; (C) E gene; (D) NS1 gene; (E) NS2A gene; (F) NS2B gene; (G) NS3; (H) NS4A; (I) NS4B; (J) NS5; (K) 3´ UTR; and (L) 5´UTR.

The ML trees constructed on the basis of individual structural and non-structural genes indicated that topologies of the structural genes and NS4A were dissimilar to the complete genome nt, aa, and ORF-based trees, either in terms of bootstrap support for different groups of DENV-2 isolates, or distribution of the isolates into various groups. The topologies of the NS5, NS3, NS1, and NS2A-based phylogenetic trees were relatively closer to the whole-genome or ORF trees than all other non-structural or structural genes. However, a clear distinction could be made between C-IA (South Asian) and C-IB (Southeast Asian) genes with considerably high (>95) bootstrap support, except for the structural genes (C, PrM/M, E) and NS4A, where the bootstrap support was not significant (<90), even though the groups were consistent (Fig 4A–4J).

DENV-2 C-II and C-III grouped separately from both C-IA and C-IB in the ML trees of all genes with high (>70) bootstrap support, except for the C gene, where the support was lower (<70) (Fig 4A–4J).

Analysis of the ML trees revealed that NS1, NS2A, NS2B, NS3, and NS5 genes were distinct in most of the Asian and Asian-American types with high bootstrap support (>70), while support for the same groups was lower (<70) in the case of NS4A and the structural genes (Fig 4A–4J). Moreover, the Asian-I DENV-2 isolate (Thailand-1964 #GQ868591) either grouped with the Asian-American types (Fig 4H; ML tree of NS4A) or formed a distinct cluster with a bootstrap support of >90 in the case of the PrM/M gene (Fig 4B). Similarly, an Asian-II isolate (Indonesiona-1975 #GQ398268*)* was found at a distinct position in the case of the NS4A ML tree (Fig 4H).

### Phylogenetic analysis based on the UTR regions

Phylogenies based on the 5´ UTR or 3´ UTR from all 50 isolates was not informative about the distribution of various DENV-2 types, as the isolates changed their positions among various groups containing different DENV-2 isolates (Fig 4K and 4L). The reason for this is that the actual sequence of the 5´ and the 3´ UTRs is unknown, because of the use of conserved primers in sequencing by the scientists who submitted the sequences to GenBank.

### Diversity among DENV-2 isolates from Pakistan

Analysis of the nine whole-genome DENV-2 sequences from Pakistan revealed that they have recently evolved from the Sri Lankan isolates and have formed a unique and distinct pattern compared to the rest of the DENV-2 isolates. All Pakistani DENV-2 isolates except one (Pak-K-2008, Accession # KF041236) clustered in the C-IA group with bootstrap support between 90 and 100, based on complete genome nt, aa, or ORF, as well as individual gene-based phylogenies (Figs 1–4J). Seven group-specific patterns of aa mutations (Table 3) were observed in the structural (PrM/M and E) and non-structural (NS1, NS2A, and NS5) genes (Table 4) of the Pakistani isolates in the C-IA subgroup, which do not exist in other DENV-2 isolates reported worldwide.

## Discussion

Previously, individual gene sequences of DENV-2 have been used for phylogenetic analysis in order to group them into various genotypes [1, 7, 21, 25, 37]. Most of the studies used E gene sequences for genotyping of DENV-2 [7, 12, 26, 27, 38, 39, 40, 41]; although, other genes have been used by some investigators [1, 11, 22, 25, 29, 42]. Based on the sequences of the E gene, DENV-2 has been divided into 5 genotypes: Cosmopolitan, Asian-I, Asian-II, Asian-American and American [7, 37]. Previous studies suggested that either the PrM/M, E, NS1, NS3, NS4A, and NS5 genes [1], or the ORFs [25], were suitable for the genotyping of DENV-2; however, these studies either did not use a phylogeny based upon whole-genome nt or aa sequences for validation of their result, or used only a limited number of local isolates in a specific country. For example, the most recent study [25] used only nine Chinese whole-genome DENV-2 sequences, or their partial gene sequences, for phylogenetic analysis encompassing only mainland China.

In our study, for the first time, 50 complete genomes of DENV-2 isolates reported from geographically distinct regions of the world were chosen for phylogenetic analysis with whole-genome sequences (both nucleotide and amino acid), ORFs, complete sequences of individual genes, and 5´ or 3´ UTRs. In addition, group-specific aa mutations prevalent in various groups of DENV-2 isolates were also observed that had not been reported in previous studies. Our results showed that the recently evolved Pakistani DENV-2 isolates form a separate and distinct subgroup (C-IA) within the main Cosmopolitan genotype, supported by a bootstrap value of 100. Similarly, other individual genes also demonstrate the existence of a distinct subgroup of Pakistani isolates, except for the E, C and NS4A genes, where the bootstrap support was less than 50. However, several investigators have previously used structural genes for the genotyping of DENV-2 isolates [7, 11, 12, 21, 26, 27, 29, 38, 39, 40].

Seven group-specific amino acid mutations in the PrM/M, E, NS1, NS2A, and NS5 genes of Pakistani isolates (C-IA) also differentiated them from the rest of the Cosmopolitan DENV-2 isolates. The C-IB subgroup did not have a group specific amino acid mutation, but as a part of the C-I group (i.e., both C-IA and C-IB), it shared 14 group-specific mutations that distinguished the C-I group from the rest of the isolates. These results suggest that Pakistani DENV-2 isolates have diverged and are evolving distinctly, probably due to several unprecedented outbreaks of DENV-2 in Pakistan since 2005 [11, 12, 29, 43].Only one Pakistani DENV-2 isolate (originally identified in 2008 in the port city of Karachi) fell into the C-IB group, which contained isolates from India, China, and Sri Lanka. There is no recent genetic evidence for the propagation of similar isolates in Pakistan during any recent outbreaks of dengue in Karachi, Lahore, or Swat, Pakistan [12, 43]; whereas, genetically similar types have been reported in India and China (Figs 1–4J).

A previous study describing the phylogenetic relationship of the Indian DENV-2 isolates [22] reported on the prevalence of a distinct South-Asian DENV-2 clade in India, which is consistent with our results for the C-IB group that contains all the Indian isolates clustered with isolates from South Asia and Southeast Asia. However, that study [22] used only E gene or ORF sequences for its phylogeny. Results obtained in our study confirmed the unique identity of the South Asian DENV-2 isolates (C-IB) by using whole-genome nt, aa, and ORFs, or all of the individual genes.

Previously, Picket et al. [44] reported 19 mutations at various positions in the NS1 gene among various DENV serotypes. However, the seven mutations we identified in the NS1 gene are different from those reported by Picket et al. [44]. In our study, type-specific patterns of amino acid substitutions were identified among various groups as diagnostic markers in order to provide further support for the existence of various groups or genotypes. Substitutions that were consistent within a single group, and not shared by other groups or genotypes, may be helpful in understanding particular evolutionary trends in a region and useful for differential diagnosis. Southeast Asian, East Asian, Australian, and African Cosmopolitan isolates also made up two distinct groups (C-II and C-III) based on whole-genome nt or aa sequences, ORFs, or individual gene-based phylogenies. The existence of the two groups was supported with a bootstrap value between 70 and 100 on the basis of the whole-genome nt and aa trees, as well as all the individual genes (Figs 1–4J). Isolates in C-III contained six group-specific amino acid mutations in the C, PrM/M, NS1, and NS2A genes, with a maximum of three specific patterns found in the NS1 gene. These type-specific amino acid mutations separated them into two distinct groups (Tables 3 and 4).

Previously described Asian (Asian-I & Asian-II) and Asian-American Cosmopolitan genotypes [1, 7] also formed distinct groups based on the whole-genome nt, aa, and ORF-based phylogenetic trees with high bootstrap support (>90). However, the bootstrap values of individual genes varied with <70 for structural genes and >90 for nonstructural genes (Fig 4A–4J), indicating that some of the structural genes reflect the same evolutionary trend and distribution as the Asian and Asian-American types, as was observed in the whole-genome phylogeny.

Although many investigators have used the E gene for the genotyping of DENV-2 isolates [7, 12, 26, 27, 37, 38, 39, 40, 41], our study indicates that this may not be suitable for the genotyping of geographically distinct DENV-2 isolates. The phylogenetic tree based on the E gene showed low bootstrap values (>30) among the American, Asian, and Asian-American types, as well as the Cosmopolitan groups. Similar results have been reported recently for Chinese isolates based on the E gene [25]. A striking feature of the ML trees was the group-displacement of the previously described Asian-I DENV-2 isolate (Thailand-1964 #GQ868591), which either grouped with the Asian-American types (Fig 4H) or claimed a distinct place with a bootstrap support of >90, based on the PrM/M tree (Fig 4B). Similarly, an Asian II isolate (Indonesia-1975 #GQ398268) was found at a distinct position in the ML tree based on the NS4A gene (Fig 4H). Displacement of these Asian-I and Asian-II genotypes in the ML trees of NS4A and PrM/M genes indicates that they may have gone through recombination events in some geographical locations and may no longer be usable for typing of the Asian genotypes, as has been reported earlier for dengue virus [45]. Among the Asian genotypes (Asian-I & Asian-II), five distinct group-specific amino acid patterns were observed in Asian-I, while the Asian-American types had a total of 10 type-specific patterns that effectively separated them into two separate groups (Tables 3 and 4). Although evolutionary distances recorded over time in the NS4A gene (Table 2) were more than those of the structural genes, the lowest number (two) of group-specific amino acid mutations was observed in the same gene among the American isolates (Table 4).

The whole-genome phylogeny divided Asian and American DENV-2 isolates into separate groups with high bootstrap support (>98), while individual gene phylogenies of C, E, NS4A, and NS4B genes also revealed the same groups but with lower support (>70). Interestingly, the American genotypes always formed a separate group and had the maximum number of group-specific amino acid mutations distinguishing them from all other groups. Nine unique patterns of aa mutations were observed solely in the NS5 genes of American isolates, indicating that this particular gene has gone through extensive selection pressure over time. This might be one of the reasons for the lower degree of fitness and subsequent replacement of the American types by the Asian-American DENV-2 (Tables 3 and 4) [8, 10, 26, 41]. Individual full-length gene phylogenies revealed that the NS5, NS3, NS1, and NS2A genes reflect comparatively similar evolutionary trends, as well as the same distribution of DENV-2 isolates with high (>90) bootstrap support as observed on the basis of whole-genome nt, aa, or ORF trees. These could therefore potentially be used for genotyping (Fig 4J, 4G, 4D and 4E). However, all structural genes and one non-structural gene (NS4A) had considerably different topologies of ML trees than did their whole-genome nt or aa trees, and thus do not seem to be suitable for the genotyping of DENV-2 or for finding evolutionary relationships among the isolates.

Among the 50 isolates of DENV-2, maximum evolutionary distances were observed in the NS2A gene, followed by the NS4A, NS4B, NS2B, NS1, and NS3 genes, which are all non-structural and important with respect to various enzymatic functions needed during the viral life cycle (Table 2). With the exception of the American isolates, mean evolutionary distances in the NS5 gene were similar to the structural genes (C, PrM/M, and E), suggesting comparatively less evolutionary pressure on the NS5 gene over time. It is possible that structural integrity of the NS5 gene is essential in viral replication of DENV-2 isolates. The maximum number of group-specific mutations was also detected in the NS5 gene, which distinguished the C-IA, C-IB, Asian-American, and American types. The majority of the group-specific mutations were found in the American isolates, which have long since been replaced with other DENV-2 isolates. These group-specific mutations could therefore be used as an important tool for the molecular detection and typing of individual isolates; however, mining of sequencing data from various geographical regions of the world on a much larger scale is needed to devise more accurate assays.

## Conclusions

Our analysis of the phylogenetic trees based on complete genomes, ORFs, or individual genes indicated that whole-genome nt, aa, and ORFs are the best options for the classification of DENV-2 isolates into various genotypes or groups, which significantly supports a further subdivision of the Cosmopolitan genotype into C-I (C-IA and C-IB), C-II, and C-III subgroups. Among the individual genes, however, some full-length NS5, NS3, NS1, and NS2A genes comparatively reflect closely related evolutionary trends but do not entirely reflect the same evolutionary trends for all the groups of DENV-2 isolates. For instance, bootstrap support in the case of Cosmopolitan II and III, Asian and Asian-American types, as well as the South Asian isolates, differs between the whole-genome ML trees and the ORF ML trees.

In addition, group-specific amino acid mutations identified in this study effectively distinguish different genotypes or groups and could also be used as diagnostic tools for the identification of various DENV-2 isolates.

## Recommendations

Firstly, only complete-genome nt or aa sequences, and ORFs should be used for classification and recombination of DENV-2 isolates into genotypes or groups, due to the lower predictive value for individual genes, but not for diagnostic purposes. For diagnostic purposes individual genes such as the NS5 gene phylogeny are sufficient for genotyping. Geographically-distinct, individual DENV-2 isolates currently grouped on the basis of individual genes should be re-assigned to their specific groups based on a complete-genome or ORF phylogeny. The use of partial sequences for determining phylogenetic relationships should be discouraged, in order to refine evolutionary trends. Group-specific patterns of amino acid mutations should be explored in other geographically-distinct DENV-2 isolates, as they could serve as valuable markers for rapid identification and typing.
